# Annexin A1 May Induce Pancreatic Cancer Progression as a Key Player of Extracellular Vesicles Effects as Evidenced in the *In Vitro* MIA PaCa-2 Model System

**DOI:** 10.3390/ijms19123878

**Published:** 2018-12-04

**Authors:** Emanuela Pessolano, Raffaella Belvedere, Valentina Bizzarro, Paola Franco, Iolanda De Marco, Amalia Porta, Alessandra Tosco, Luca Parente, Mauro Perretti, Antonello Petrella

**Affiliations:** 1 Department of Pharmacy, University of Salerno, via Giovanni Paolo II 132, 84084 Fisciano, Italy; epessolano@unisa.it (E.P.); rbelvedere@unisa.it (R.B.); vbizzarro@unisa.it (V.B.); aporta@unisa.it (A.P.); tosco@unisa.it (A.T.); lparente@unisa.it (L.P.); 2 Department of Industrial Engineering, University of Salerno, via Giovanni Paolo II 132, 84084 Fisciano, Italy; pfranco@unisa.it (P.F.); idemarco@unisa.it (I.D.M.); 3 The William Harvey Research Institute, Barts and The London School of Medicine and Dentistry, Queen Mary University of London, London EC1M 6BQ, UK; m.perretti@qmul.ac.uk

**Keywords:** Annexin A1, EVs, pancreatic cancer, CRISPR/Cas9 genome editing technique, epithelial to mesenchymal transition

## Abstract

Pancreatic Cancer (PC) is one of the most aggressive malignancies worldwide. As annexin A1 (ANXA1) is implicated in the establishment of tumour metastasis, the role of the protein in PC progression as a component of extracellular vesicles (EVs) has been investigated. EVs were isolated from wild type (WT) and ANXA1 knock-out (KO) PC cells and then characterised by multiple approaches including Western blotting, Field Emission-Scanning Electron Microscopy, and Dynamic Light Scattering. The effects of ANXA1 on tumour aggressiveness were investigated by Wound-Healing and invasion assays and microscopic analysis of the Epithelial to Mesenchymal Transition (EMT). The role of ANXA1 on angiogenesis was also examined in endothelial cells, using similar approaches. We found that WT cells released more EVs enriched in exosomes than those from cells lacking ANXA1. Notably, ANXA1 KO cells recovered their metastatic potential only when treated by WT EVs as they underwent EMT and a significant increase of motility. Similarly, human umbilical vein endothelial cells (HUVEC) migrated and invaded more rapidly when treated by WT EVs whereas ANXA1 KO EVs weakly induced angiogenesis. This study suggests that EVs-related ANXA1 is able to promote cell migration, invasion, and angiogenesis, confirming the relevance of this protein in PC progression.

## 1. Introduction

Annexin A1 (ANXA1) is a 37-kDa protein belonging to the annexin superfamily and is able to bind membrane phospholipids in a calcium-dependent manner [1]. Besides the large spectrum of physiological functions, rising evidences implicated ANXA1 in the process of carcinogenesis in a tumour-specific manner [2,3]. It is known that ANXA1 is differentially expressed in various tumours and it might specifically function as either suppressor or promoter of neoplastic development [4]. ANXA1 has different roles according to its subcellular localization. Particularly, its activity could be mediated by both the secreted and the intracellular forms. In the first case, the protein is able to bind the G-protein coupled formyl peptide receptor (FPRs) family that is involved in cell motility; in the second one, it directly and indirectly promotes cytoskeletal organization [5,6,7]. Thus, ANXA1 has the potential role of biomarker for diagnosis, treatment, and prognosis of certain tumours.

Among the several tumour models where the role of ANXA1 remains controversial, in pancreatic cancer (PC) ANXA1 expression is related to tumour development and drug resistance [8,9,10]. Recent studies have shown that the ANXA1 strongly affects migration and invasion of PC cells and is involved in the metastatization process. Particularly, thanks to the establishment of ANXA1 knock-out (KO) MIA PaCa-2 PC cells through the CRISPR/Cas9 genome editing system, it has been possible to show that the protein is able to trigger the epithelial to mesenchymal transition (EMT), leading to a more aggressive phenotype [7,11,12]. 

The EMT is characterized not only by the signal transduction networks and transcriptional factors but also by secreted proteins and membrane-derived extracellular vesicles (EVs) [13]. EVs are a heterogeneous group of macro- and micro-vesicles. The latter includes small EVs like exosomes, ectosomes, apoptotic bodies, and oncosomes, differentiated by size and biogenesis mechanisms. In particular, exosomes are nanosized vesicles in a range of 40–150 nm, with a still unclear biogenesis that has been recognized as bimodal actors in tumour progression [14]. On one hand EVs may program the immune system to elicit an anti-tumour response, on the other hand they could function as a long-distance communication tool by which tumour cells may form pre-metastatic niches in secondary organs [15]. Thus, in the last decade, EVs have been considered as both putative targets in cancer, affecting metastatic cascade, and vehicles for drug delivery in the area of the nanomedicine [16,17]. In the PC model, EVs could be useful as tool for screening, diagnosis, and prognosis for the early detection of the disease [18]. Encouraging results highlighted that EVs with exosomal features promote the aggressive behaviour of PC, leading to recurrence and metastasis [19]. 

As mentioned above, ANXA1 could be secreted in the extracellular environments to carry out some of its functions. However, the mechanisms of protein secretion are not well defined to date. ANXA1 could be externalized through direct interaction with the plasma membrane or membrane transporters. Furthermore, the protein plays an important intracellular role in vesicle-trafficking, mediating the interaction of vesicles with cytoplasmic machinery, particularly for the inward vesiculation process [20]. Notably, in the field of cancer, the EV pathway may represent the main mechanism of ANXA1 externalization, evolving through the exocytosis of microvesicles by fusion of multivesicular endosomes with the plasma membrane [3]. Nevertheless, high levels of ANXA1-containing EVs have been found in the serum of patients affected by intestinal mucosa inflammation and in cases of more aggressive forms of glioblastoma [21,22].

The roles of ANXA1 in EV biogenesis and on PC progression as a part of EV machinery are not yet documented. Thus, we investigated, for the first time, the role of secreted ANXA1 as a component of small vesicle contents from MIA PaCa-2 PC cells. To test the protein impact on the tumour aggressiveness, we analyzed the behaviour of wild type (WT) and/or ANXA1 KO MIA PaCa-2 cells, treated with EVs-depleted and EVs-associated, enriched in exosomes, fractions from recipient cells. Moreover, we investigated the activation of endothelial cells evaluating the pro-angiogenic effects of ANXA1 contained in EVs from PC cells.

## 2. Results

### 2.1. Characterization of EVs Released from WT and ANXA1 KO MIA PaCa-2 Cells

ANXA1 has been previously associated with the content of EVs in various type of cancer [23,24]. This suggests that the protein could have a role in both the formation of these vesicles and in EV-related tumour progression and metastatic development [7]. 

**Figure 1:**
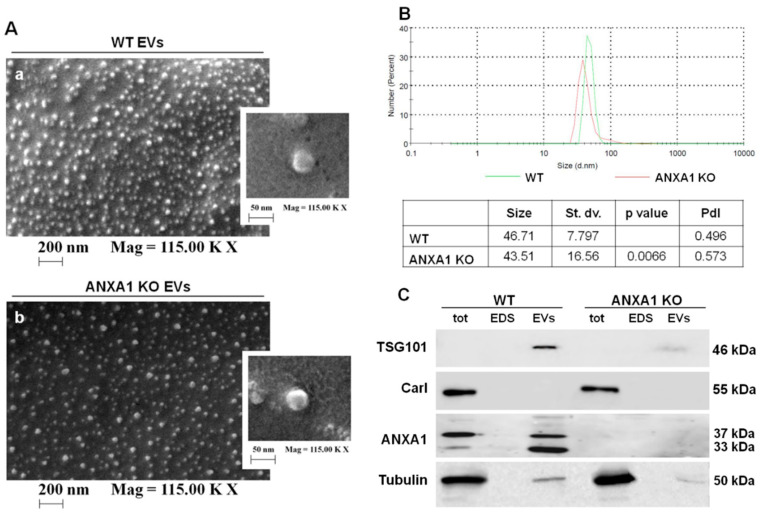
Analysis of extracellular vesicles (EVs) deriving from wild type (WT) and annexin A1 (ANXA1) knock-out (KO) MIA PaCa-2 cells. (**A**) EVs isolated from WT (a) and ANXA1 KO (b) MIA PaCa-2 cells were imaged by Field Emission Scanning Electron Microscopy (FE-SEM). Magnitude = 11,500 KX (that is 115,000) and scale bar = 200 nm. For the magnification: magnitude 11,500 KX (that is 115,000) and scale bar = 50 nm (**B**) Characterization of the EVs size distributions by dynamic light-scattering (DLS). The table reports the EVs mean of size, standard deviation, *p* value, referring to ANXA1 KO EVs distribution vs. WT, and PdI. The experiments were performed in triplicate. (**C**) Western blot using antibodies against TSG101, calreticulin, and ANXA1 on protein content of total cell lysates, EV-depleted extracellular fractions (EDS) and EVs fractions extracted from WT and ANXA1 KO MIA PaCa-2 cells. Protein normalization and the check of the sample quality were performed on tubulin levels.

In this study, tumour-derived EVs were initially purified from the supernatant of both WT and ANXA1 KO MIA PaCa-2 cells. The purified EVs were examined by Field Emission Scanning Electron Microscopy (FE-SEM), which showed typical rounded particles ranging from 30 to 180 nm in diameter (Figure 1A panels a–b). Interestingly, we found that ANXA1 KO MIA PaCa-2 cells appeared to release fewer EVs than WT MIA PaCa-2 cells (Figure 1A panels a–b). This result has been confirmed by dynamic light-scattering (DLS) analysis, by which we estimated the EVs size distribution by number depending on the measurement of the diameter. In the graph of Figure 1B the green curve refers to WT and the red one to ANXA1 KO MIA PaCa-2 cells. The different heights show the EVs relative yield. The table reports the mean of size, the standard deviation (St. dv.), the p value, referring to ANXA1 KO EVs distribution versus WT one, and the Polydispersity Index (PdI) deriving from DLS. The 99.5% and the 99.9% of the values derived from the analysis of WT and ANXA1 KO EVs samples, respectively, are in the area under the curves. Among these values, ranging from about 20 to 100 nm, 37% have a mean diameter of 46.71 nm for WT cells and 28% have a mean diameter of 43.51 nm for ANXA1 KO ones.

In order to analyse the involvement of ANXA1 in EV biogenesis, we also performed Western blot analysis on vesicles extracted from WT and ANXA1 KO MIA PaCa-2 cells. 

Among the variety of proteins that are contained in EVs, particularly exosomes, TSG101 protein is the main characterized and the mostly used marker [25]. Therefore, the presence of the protein in the EVs extracted from WT and ANXA1 KO MIA PaCa-2 cells was investigated. As shown in Figure 1C we found that TSG101 was expressed only in EV fractions whereas it was absent in whole cell lysates (tot) and in EV-depleted extracellular fractions (EDS), all prepared as reported in Materials and Methods section.

In parallel, the expression of calreticulin frequently used as negative control marker for EVs was investigated. This protein is exposed on the surface of apoptotic cells and ends up in apoptotic bodies, while not being enriched in EVs [26]. In the Western blot analysis, the calreticulin appeared only in total cell lysates from WT and ANXA1 KO MIA PaCa-2 cells while no signals were observed in EDS and EVs fractions.

Finally, we confirmed the effective presence of ANXA1 in whole lysates and in the EVs isolated by MIA PaCa-2 WT cells by Western blot analysis (Figure 1C). Notably, in EV fractions from WT MIA PaCa-2 cells the 33 KDa cleaved form of ANXA1 protein was detected in addition to the 37kDa full-length form. The absence of ANXA1 expression was established in all the extracts from ANXA1 KO MIA PaCa-2 cells (Figure 1C). 

Thus, we found that WT MIA PaCa-2 cells secreted a greater amount of EVs which contain the largest part of secreted ANXA1. 

### 2.2. EVs Isolated from WT MIA PaCa-2 Cells Increase Cell Migration and Invasion Rate

It is known that ANXA1 in PC progression [8,9]. In previous experiments, we observed that ANXA1 KO MIA PaCa-2 cells have a weak motile phenotype if compared with that of WT MIA PaCa-2 cells [11]. Particularly, the extracellular form of ANXA1 plays an important role in cancer cell migration, invasion and metastasis [7].

**Figure 2:**
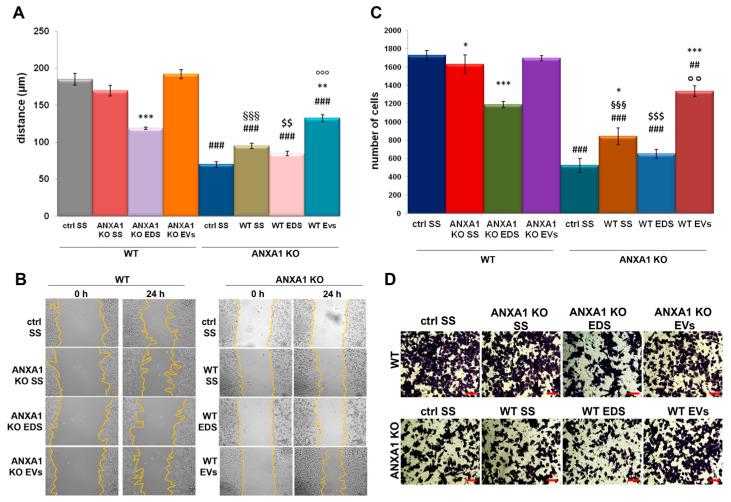
EVs released by WT and ANXA1 KO MIA PaCa-2 affected cell migration and invasion rate. Effects of starved supernatants (SS), EDS, and EVs fractions from MIA PaCa-2 and ANXA1 KO MIA PaCa-2 on WT and ANXA1 KO recipient cells and viceversa in Wound-Healing (**A**) and invasion (**C**) assays. Representative images for migration and invasion assays are reported in (**B**) and (**D**), respectively. Bar = 50 µm. Statistical significance was calculated using *t*-test, * (asterisk) *p* < 0.05, ** *p* < 0.01, *** *p* < 0.001 treated cells vs untreated controls; ## *p* < 0.01 and ### *p* < 0.001 for each point of ANXA1 KO MIA PaCa-2 cells vs WT one, §§§ *p* < 0.001 for the experimental points treated with ANXA1 KO cell SS vs WT MIA PaCa-2 one, $$ *p* < 0.01, $$$ *p* < 0.001 for cells treated with ANXA1 KO MIA PaCa-2 EDS vs WT MIA PaCa-2 EDS and °° (circlets) *p* < 0.01 and °°° *p* < 0.001 for cells in presence of EVs secreted by ANXA1 KO MIA PaCa-2 vs EVs from WT MIA PaCa-2.

To characterize the contribution of ANXA1-containing EVs on tumour aggressiveness, we compared the effects of the addiction of whole serum starved supernatants (SS), EV-depleted (EDS), and EV-enriched (EVs) fractions from both WT and ANXA1 KO MIA PaCa-2 cells on their own motility potential (Figure S1) and by adding the fractions from WT source cell line to ANXA1 KO recipient cells and viceversa (Figure 2A–D).

First, we analysed by Wound Healing assay the migratory behaviour of WT and ANXA1 KO MIA PaCa-2 cells in the presence of all extracellular fractions from both cell lines inverted as previously described, focusing on the effects of SS, EDS, and EVs fractions containing or not ANXA1 (Figure 2A,B). Figure 2A shows that ANXA1 KO cells underwent a significant increase of their migration rate when treated by EVs fractions isolated by WT MIA PaCa-2 cells while SS and EDS fractions derived from the same cell source only partially affected ANXA1 KO MIA PaCa-2 motility (Figure 2A,B). Notably, no significant effects on cell migration were observed in WT MIA PaCa-2 treated by all fractions obtained from ANXA1 KO PC cells with the exception of the EDS fraction that partially reduced cell migration (Figure 2A,B) suggesting a key role of ANXA1 in migration related events. 

No significant effects on cell migration were observed when WT MIA PaCa-2 and ANXA1 KO MIA PaCa-2 cells were treated by their own fractions except for WT EDS and EVs, and ANXA1 KO EVs that partially induced cell migration (Figure S1A).

Similarly, SS and mainly EVs fractions isolated by WT MIA PaCa-2 cells, and therefore containing ANXA1, considerably increased the invasion ability of ANXA1 KO MIA PaCa-2 cells as shown in Figure 2 panels C and D. No significant effects on cell invasion were observed in cells treated by EDS fractions (Figure 2C,D). The SS and EDS fractions from ANXA1 KO MIA PaCa-2 cells were able to affect the invasive behaviour of the WT cells by reducing the invasion rate partially with SS while strongly with EDS fractions (Figure 2C,D). No significant effects on cell invasion were observed in WT MIA PaCa-2 and ANXA1 KO MIA PaCa-2 cells treated by their own fractions except for EVs that in both cases induced the increase of cell invasion rate (Figure S1B).

In conclusion, we confirmed that extracellular ANXA1 is able to positively affect migration and invasion processes but we also showed that the main function is carried out by its form contained in EVs.

### 2.3. ANXA1-Containing EVs Induce a Switch of Phenotype in PC Cells

**Figure 3:**
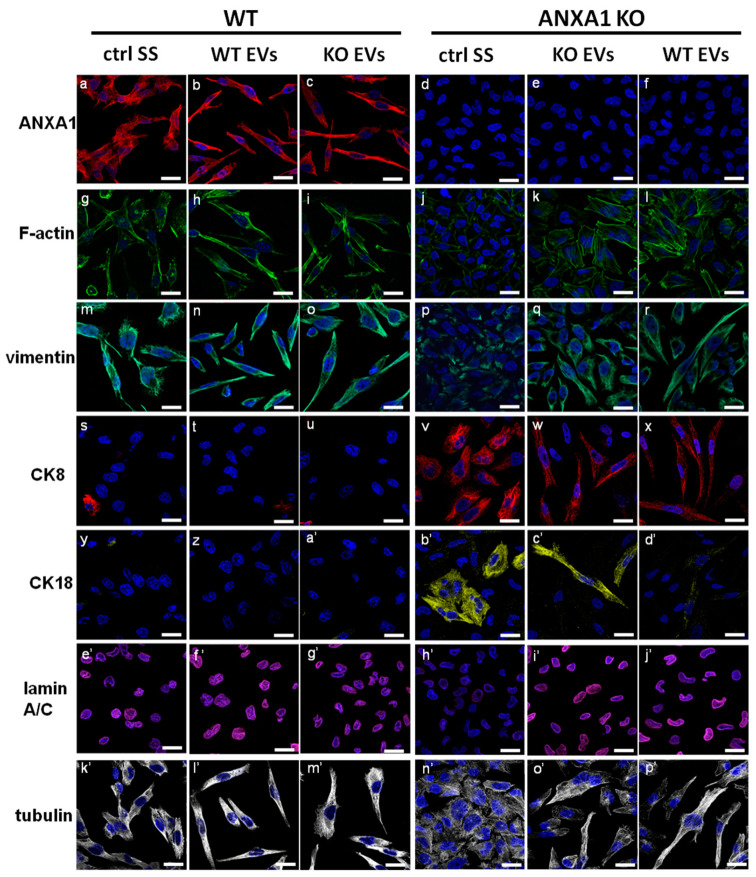
Evaluation of the main Epithelial to Mesenchymal Transition (EMT) markers on WT and ANXA1 KO MIA PaCa-2 cells in presence of EVs. Immunofluorescence analysis to detect: ANXA1 (panels **a**–**c** for WT and d–f for ANXA1 KO cells), F-actin (panels **g**–**i** for WT and **j**–**l** for ANXA1 KO), vimentin (panels **m**–**o** for WT and **p**–**r** for ANXA1 KO cells), CK8 (panels **s**–**u** for WT and **v**–**x** for ANXA1 KO cells), CK18 (panels **y**,**z**,**a’** for WT and **b’**–**d’** for ANXA1 KO cells), lamin A/C (panels **e’**–**g’** for WT and **h’**–**j’** for ANXA1 KO cells) and tubulin (panels **k’**–**m’** for WT and **n’**–**p’** for ANXA1 KO cells) on WT and ANXA1 KO MIA PaCa-2 cells, treated or not with EVs released from both cell lines. Nuclei were stained with Hoechst 33342 1:1000 for 30 min at room temperature (RT) in the dark. Magnification 63× 1.4 numerical aperture (NA). Bar = 10 µm.

Extracellular ANXA1 has been described to play several roles in the acquisition of a more aggressive phenotype in cancer cells, for example as a modulator of EMT-like phenotypic switch [6,7]. However, very little is known about its role as a component of EVs released from cancer cells on EMT process. Thus, we investigated by confocal microscopy (Figure 3) the effects of EVs from WT and ANXA1 KO PC cells on morphological features and on the expression of some proteins involved in EMT in PC recipient cells. WT and ANXA1 KO MIA PaCa-2 cells were treated with both their own and inverted different fractions of cell-conditioned or not-conditioned supernatants (SS, EDS and EVs), as reported in Materials and Methods section. 

First, the effects on ANXA1 expression of EV addition on WT and ANXA1 KO PC cells was analyzed. We found that EVs were ineffective on protein expression as well as all the supernatant fractions (Figure 3a–f; Figure S2a–e; Figure S3a–e). Notably, in these cells a significant modification of morphology was observed as both WT and ANXA1 KO cells appeared significantly elongated in all experimental conditions (Figure 3 and Figures S2 and S3). On the other hand, F-actin and tubulin staining showed a well organized cytoskeleton (Figure 3k’–p’) with the appearance of several stress fibres (Figure 3g–l) only in the cells treated by fractions from WT cells and partially by EVs from ANXA1 KO cells, suggesting the occurrence of a more motile phenotype and highlighting the role of ANXA1 in this event.

Contextually, we found the increase, as well as a better filamentous-like organization, of the EMT protein vimentin in ANXA1 KO cells (Figure 3p,r) treated by EVs fractions from WT MIA PaCa-2 cells where a loss of the epithelial cytokeratins 8 (CK8) (Figure 3, panels v,x) and 18 (CK18) (Figure 3b’,d’) was also observed. EVs from ANXA1 KO cells were able to partially induce vimentin increase (Figure 3p,q) and CK8/CK18 decrease (Figure 3v,w,b’,c’). No differences were found in vimentin, CK8 and CK18 expression in WT PC cells treated by the other fractions from ANXA1 KO cells (Figure 3, panels m–o; panels s–u; panels y–a’) whereas SS and EDS fractions from WT MIA PaCa-2 were able to partially induce both vimentin increase and CK8/18 decrease in ANXA1 KO cells (Figure S3k–o, p–t, u–y).

Finally, lamin A/C, a protein associated with cytoskeletal dynamics and weakly expressed in low aggressive PC [27] as well as in ANXA1 KO PC cells [11], started to be expressed in ANXA1 KO MIA PaCa-2 only when these cells were treated by EDS and EVs fractions from WT cells (Figure 3e’–j’; Figure S3z–d’). Even in this case, no differences were found in lamin A/C expression in WT PC cells treated by all fractions from ANXA1 KO cells (Figure 3m–o, s–u, y–a’). The quantification of the fluorescence intensity of the protein shown in Figure 3 is reported in Figure S4.

These results showed the ability of ANXA1 contained in EVs to induce the EMT in ANXA1 KO MIA PaCa-2 cells. 

### 2.4. Effects of EVs on Endothelial Cell Activation

**Figure 4:**
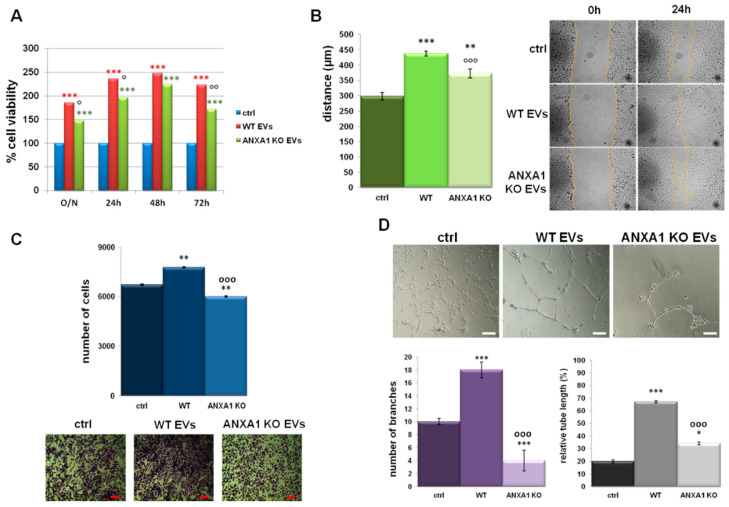
Effects of WT and ANXA1 KO MIA PaCa-2-derived EVs on HUVEC cells. (**A**) MTT (3-(4,5-Dimethylthiazol-2-yl)-2,5-Diphenyltetrazolium Bromide) assay on HUVEC evaluating cell viability. Results of Wound-Healing assay (**B**) and analysis of invasion speed with relative bright field images (**C**) of HUVEC cells treated or not with EVs from WT and ANXA1 KO MIA PaCa-2 cells. Bar = 50 µm. (**D**) Representative images of tube formation by HUVEC cells seeded for 12 h on matrigel: EBM-2 1:1 and treated or not with EVs from WT and ANXA1 KO MIA PaCa-2 cells. Analysis of tube length and number of branches calculated by ImageJ (Angiogenesis Analyzer tool) software. Bar = 100 µm. Data represent the mean of three independent experiments ± standard deviation with similar results, ° *p* < 0.05, *** (red and black asterisk) *p* < 0.001 treated cells vs untreated control and °°° (circlets) *p* < 0.001 for HUVEC cells treated with ANXA1 KO MIA PaCa-2 EVs vs WT MIA PaCa-2 EVs.

Intra- and extracellular ANXA1 has been described as a pro-angiogenic protein [28,29]. Thus, the paracrine effects of EVs derived by WT and ANXA1 KO MIA PaCa-2 cells on endothelial cells was investigated analysing the increase of proliferation rate of HUVEC (Human Umbilical Vein Endothelial Cells) in the presence of the EVs fractions from WT MIA PaCa-2 cells. These EVs were able to induce a stronger proliferative effect than those from ANXA1 KO MIA PaCa-2 cells (Figure 4A). Furthermore, as shown in Figure 4B,C, HUVEC cells migrated and invaded the matrigel coating more rapidly in the presence of EVs from WT MIA PaCa-2 compared to those isolated from ANXA1 KO MIA PaCa-2 cells. Nevertheless, in both cases treated cells were faster than not treated controls. Once evaluated the migration and the invasion as starting processes of the angiogenesis, the *in vitro* tubulogenesis was finally studied. HUVEC cells were able to form capillary-like structures when treated with EVs deriving from WT MIA PaCa-2 cells more than with those obtained from ANXA1 KO cells. These events were evident in the bright field images and were further corroborated by the analysis of the number of branching points and of the relative tube length calculated as reported in the Material and Methods section (Figure 4D).

All the technical controls are reported in Figure S5. We replaced the HUVEC growth medium with the 25% of the total volume of WT and ANXA1 KO MIA PaCa-2 cell complete (sup) and SS supernatants, and with the EDS derived from the same cell lines. In this supplementary figure we show the results obtained from the *in vitro* Wound-Healing (Figure S5A), invasion (Figure S5B), and tube formation (Figure S5C) assays. sup, SS and EDS fractions from WT MIA PaCa-2 cells induced a significant increase of migration and invasion rate. On the contrary, those ones deriving from ANXA1 KO MIA PaCa-2 cells did not affect these processes. The formation of capillary-like structures resulted particularly increased in presence of the complete supernatant of WT MIA PaCa-2 cells, including both FBS and the soluble form of ANXA1. The analysis of relative tube length and of the number of branching points showed that the absence of a part of growth factors in HUVEC medium was an important limiting issue for tube formation, independently of the single specific treatment. 

The activation of endothelial cells by EVs obtained from WT MIA PaCa-2 cells not only indicated their impact on tumour progression but also confirmed the role of ANXA1 in this process.

## 3. Discussion

EVs are involved in the dissemination of cancer. These vesicles, mainly exosomes, when derived from tumour cells, could promote the migration and the invasion speed of the same cells as well of the cells of surrounding tissues [30]. Indeed, EVs affect cellular physiology also by triggering vascular permeability or by conditioning pre-metastatic sites in distant organs, in autocrine and paracrine manners [31]. The role of EVs is not fully understood for many cancer models but in PC it is reported that these microvesicles can themselves migrate to distant organs and promote the formation of pre-metastatic niches [17]. Exosome proteomic analysis performed by Yu et al., identified ANXA1 as one of the proteins associated to PC metastasis in multiple organs, mostly in liver [32]. Furthermore, our previous work has shown that ANXA1 favours PC metastasis acting in the intracellular environment as a cytoskeleton remodelling factor. The extracellular form also promotes metastasis acting either as agonist of FPRs or independently of the receptor activation [7,11].

As ANXA1 can be considered as a PC diagnostic/prognostic marker, it is important to understand if its mechanism could be related to EVs secretion and/or activity. In this study, we investigated the role of ANXA1 in the metastatic potential of EVs released from MIA PaCa-2 PC cells. 

Firstly, the characterization of the EVs, enriched in exosomes, isolated by our *in vitro* model, revealed the importance of ANXA1 in the production and/or secretion of these microvesicles. The finding of a lower quantity of EVs in the ANXA1 KO MIA PaCa-2 media supports the concept that the protein participates to vesicle trafficking [20,33]. It is well known that in the inward vesiculation process ANXA1 could form a bridge between the membranes of cell and vesicle through the amphipathic N-terminal helix. In other cases, two ANXA1 molecules on opposing membranes could dimerize and form a heterotetramer with two molecules of S100A11 [34]. EVs, particularly exosomes, are formed by intraluminal budding within multivesicular bodies which undergo a fusion with the plasma membrane through endocytosis and exocytosis events [35]. Thus, it can be hypothesized that there is involvement of ANXA1 also in the outward vesiculation, creating a bridge between the two directions. Moreover, the presence of both 37 and 33 kDa ANXA1 forms in EVs from WT MIA PaCa-2 cell fractions confirms previous results [7] and suggests the requirement of post-transductional modifications, i.e. phosphorylations, followed by cleavage, to trigger ANXA1 externalization both as soluble protein and/or through EVs [21]. Indeed, these modifications are responsible for different localization, binding properties, and function of ANXA1 and can be associated to PC aggressiveness. Several biochemical approaches have revealed the importance of the cleavage for the ANXA1 functions in the inflammatory system, not yet in cancer models [36,37,38,39]. Furthermore, in EVs the protein appeared abundant near the inner surface of the microvesicles where it is probably cleaved by MMPs 2 and 9 and/or ADAM-10 [21]. However, there is no evidence which could allow researchers to speculate about the role of cleaved ANXA1 as well as all its post-transductional modifications in EVs. 

In this work we focused not only on the role of ANXA1 in EVs biogenesis, but the mechanism by which extracellular ANXA1 contributes to the PC aggressiveness in its counterpart independent of FPR activation was also investigated. 

The addition of all supernatant fractions from WT cells (SS, EDS and EVs) on ANXA1 KO MIA PaCa-2 was able to increase the rate of migration and invasion processes, highlighting the crucial role of ANXA1 in the extracellular environment to induce motility. This aspect has been also confirmed by the administration of the same amount of EVs secreted by WT and ANXA1 KO MIA PaCa-2 on recipient cells. In this way, we referred exclusively to the features of these microvesicles. Furthermore, the inability of EDS fractions obtained from WT and ANXA1 KO MIA PaCa-2 confirmed that the EVs have a dominant role in inducing these processes on PC cells. These findings suggest that extracellular ANXA1 and EVs are the two faces of the same medal for the modulation of PC progression. The cross-linking between ANXA1/EVs and PC aggressiveness has been confirmed by the analysis of EMT markers, since the acquisition of a mesenchymal phenotype appeared essential for cell spreading to distant sites [11,40]. 

Taken together, the present results could help to clarify a loop ANXA1/EVs, suggesting the participation of ANXA1 in EVs externalization through an outward mechanism of vesicles transport. Furthermore, since the ANXA1 is a part of EVs protein content, it could be endocyted or not by recipient cells. In the first case ANXA1 could act independently of FPRs, in the second one the protein can trigger the receptor activation both as full length protein and as N-terminal peptide. Actually, we did not expect the uptake of ANXA1 deriving from the EVs by recipient cells since the extra- and the intra-cellular forms of this protein are able to differently act, as reported by several works and how we previously showed [3,7,11,21,41]. These studies led to the hypothesis for which the axis ANXA1/EVs-FPRs in PC progression could have great relevance. According to this supposition, our preliminary data show that the ANXA1/EVs complex is able to activate FPRs. Thus, this is the aim of a future work with which we would like to characterize the mechanism of action of the extracellular protein in our in vitro model.

During the phases of tumour development, the cell-to-cell communication between PC and stromal cells via EVs has a pivotal role through the activation of endothelial cells and the induction of angiogenesis [42,43,44]. Hence, the targeted inhibition of EVs pro-angiogenic functions might be a novel therapeutic approach to control tumour progression [45]. Thus, in our *in vitro* models, we confirmed the activation of endothelial cells by the increase of proliferation, migration, and invasion rates, as well as the stimulation of angiogenesis in presence of EVs. Particularly, the combined effects of ANXA1 and of the remaining EV content from WT MIA PaCa-2 cells confirm that the protein enhances the EVs abilities in this process. Although the mechanism of interaction of ANXA1/EVs with target cells remains controversial [46], the evidence that the extracellular form of ANXA1 explicates its pro-angiogenic function through EVs may represent another potential target in the therapy/prevention of PC dissemination. 

This outcome highlights the effect of the loop ANXA1/EVs not only on tumour cells but also on PC surrounding tissues as indicated by the formation of the capillary-like structures from endothelial cells. Future work will examine different cell populations as fibroblasts or keratinocytes in order to identify the molecular mechanisms of this loop. Generally, some aspects as tumour microenvironment, immunity, and metastatization will be explored in *in vivo* models in order to better define the impact of ANXA1 on EVs biogenesis and of the complex ANXA1/EVs in PC progression.

## 4. Material and Methods

### 4.1. Cell Culture

MIA PaCa-2 cells were purchased from American Type Culture Collection (ATCC CRL-1420; Manassas, VA USA) and cultured in high glucose DMEM containing L-Glutamine 2 mM, 10% heat-inactivated fetal bovine serum (FBS), and 10,000 U/mL penicillin and 10 mg/mL streptomycin (Euroclone; Milan, Italy). Cells were maintained at 37 °C in 5% CO_2_-95% air humidified atmosphere. ANXA1 KO MIA PaCa-2 cells were obtained using CRISPR-Cas9 plasmid purchased from GenScript (Piscataway, NJ, USA), as reported in [11]. These clones were kept in selection by 700 μg/mL neomycin (Euroclone; Milan, Italy).

HUVEC cell line was purchased from ATCC (Manassas, VA USA) (ATCC^®^ PCS-100-010™) and was maintained as reported in [47]. Cells were cultured until passage 10.

### 4.2. Exosomes Enrichment

The enrichment of exosomes from cell culture supernatants has been performed as reported in [48]. WT and ANXA1 KO MIA PaCa-2 cells (1.5 × 10^5^ cm^−2^, for a total of about 8 × 10^7^ cells) were incubated for 24 h in DMEM medium without FBS. Conditioned medium (also reported as SS) was collected and centrifuged for 5 min at 300× *g* at room temperature (RT) to remove detached cells; the supernatant was transferred and centrifuged for 10 min at 2,000× *g* at 4 °C to remove dead cells. The obtained supernatant was transferred and centrifuged at 10,000× *g* for 30 min at 4 °C to eliminate cell debris. Then, the cleared supernatant was transferred to ultracentrifuge tubes and centrifuged for 70 min at 100,000× *g* at 4 °C. Next, the supernatant was stored and used as EDS (EVs-depleted supernatant); the pellet was washed in PBS and re-ultracentrifuged at 100,000× *g* at 4 °C for 70 min. Finally, the supernatant was removed and the pellet was resuspended. The buffer we selected for the resuspension was sterile bidistilled water with 5 mM EDTA, to avoid vesicles aggregation, for FE-SEM (Field Emission-Scanning Electron Microscope) and DLS (Dynamic light scattering) analysis, 50 µL RIPA lysis buffer for Western blotting, or 200 µL PBS for the administration to cells. To flatten the differences of amount of EVs observed by DLS technique, we used 9% more of EVs fraction from ANXA1 KO MIA PaCa-2 to treat cells. This difference was also corroborated by Bradford assay that furnished similar results and by which we calculated about 20 µg of EVs proteins from WT MIA PaCa-2 and about 21.8 µg (9% more) for those from ANXA1 KO ones. The normalization through Bradford assay has been performed using the correspondent amount of EVs lysed in RIPA buffer. This normalization has been important for us in order to administrate to cells the same amount of EVs, derived from WT and ANXA1 KO MIA PaCa-2 cells, on all the experimental points. All analyses were performed on fresh isolated fractions. 

### 4.3. Field Emission-Scanning Electron Microscope (FE-SEM) Analysis

Sample morphology was analyzed using a FE-SEM model LEO 1525 (Carl Zeiss SMTAG; Oberkochen, Germany). The EVs enriched in exosomes were fixed with 2% *v*/*v* p-formaldehyde and 1% *v*/*v* glutaraldehyde (Sigma-Aldrich; Saint Louis, MO, USA) in PBS. Next, a drop of the suspension was spread on a carbon tab placed on an aluminium stub (Agar Scientific; Stansted, UK) and left to dry in a stream of nitrogen for 25 min. Then, the dried samples were coated with gold (layer thickness 250 Å) using a sputter coater (model 108 A, Agar Scientific; Stansted, UK). Each analysis was performed in triplicate.

### 4.4. Dynamic Light Scattering (DLS) Analysis

The DLS technique was performed using Zetasizer Nano S instrument (Worcestershire, UK) in order to obtain particle size distribution by number of the EVs. The DLS instrument works at 25 °C and is equipped with a 5.0 mW He-Ne laser operating at 633 nm with a scattering angle of 173°. Each measurement was repeated in triplicate.

### 4.5. Western Blotting

Proteins extracted by cells were examined by SDS-PAGE, SS, EDS and EVs. Protein content was estimated according to Biorad protein assay (BIO-RAD, Hercules, CA, USA), as previously described [49]. 20 µg of proteins were visualized using the chemioluminescence detection system (Amersham biosciences; Little Chalfont, UK) after incubation with rabbit polyclonal primary antibodies against ANXA1 (1:10,000; Invitrogen; Carlsbad, CA, USA) and calreticulin (1:1000; Elabscience; Houston, TX, USA), with mouse monoclonal primary antibodies against TSG101 (1:1000; ThermoFisher Scientific; Waltham, MA, USA) and α-tubulin (1:1000; Sigma-Aldrich; Saint Louis, MO, USA). The blots were exposed to Las4000 (GE Healthcare Life Sciences; Little Chalfont, UK). 

### 4.6. Wound-Healing Assay

A wound was produced on the confluent monolayer of WT and ANXA1 KO MIA PaCa-2 and of HUVEC by scraping the cells with a pipette tip. Next, cells were incubated with conditioned media (sup), SS, EDS, and EVs fractions inverted or not and further treated with mitomycin C (10 μg/mL, Sigma-Aldrich; Saint Louis, MO, USA), to ensure the block of mitosis. Each experimental point was treated without FBS, including the control. The wounded cells were analyzed as reported in [11]. The reported values represent the mean of the measured distances of five different positions for which ten cells were selected on the two sides of wound. The data are representative of five experiments with similar results. 

### 4.7. Invasion Assay

WT and ANXA1 KO MIA PaCa-2 and HUVEC invasiveness was studied using the trans-well cell culture (12 mm diameter, 8.0-fim pore size) purchased form Corning Incorporated (New York, NJ, USA), as previously described [50]. In the lower chamber of each well were added SS, EDS, and EVs fractions from WT or ANXA1 KO MIA PaCa-2 cells as inverted media or not as technical controls. At 24 h after seeding and treatments, included the mitomycin C (10 μg/mL, Sigma-Aldrich; Saint Louis, MO, USA) to ensure the block of mitosis. The procedures of staining and analysis were performed as reported in [12]. 

### 4.8. MTT Assay

HUVEC cells were harvested at the indicated times (overnight, 24, 48 and 72 h) in presence or not of treatments. Cell viability was calculated as previously described [6]. The optical density (OD) of each well was measured with a spectrophotometer (Titertek Multiskan MCC/340) equipped with a 620 nm filter.

### 4.9. Confocal Microscopy

WT and ANXA1 KO MIA PaCa-2, fixed in p-formaldehyde (4% *v*/*v* in PBS; Lonza; Basilea, Swiss), were permeabilized or not with Triton X-100 (0.5% *v*/*v* in PBS; Lonza; Basilea, Swiss), blocked with goat serum (20% *v*/*v* in PBS; Lonza; Basilea, Swiss) and then incubated with anti-ANXA1 antibody (rabbit polyclonal; 1:100; Invitrogen; Carlsbad, CA, USA), anti-vimentin (mouse monoclonal, 1:500; Santa Cruz Biotechnologies; Dallas, TX, USA), anti-CK8 (mouse monoclonal, 1:200; Santa Cruz Biotechnologies; Dallas, TX, USA), anti-CK18 (mouse monoclonal, 1:200; Santa Cruz Biotechnologies; Dallas, TX, USA) anti-lamin A/C (mouse monoclonal, 1:450; Novocastra; Wetzlar, Germany) and α-tubulin (1:100; Sigma-Aldrich; Saint Louis, MO, USA) O/N at 4 °C. F-actin was evaluated by 5 µg/mL of Phalloidin-FITC (Sigma-Aldrich; Saint Louis, MO, USA) for 30 min at RT in the dark. The staining with the anti-rabbit and anti-mouse antibodies and for the nuclei and the following confocal analysis were performed as reported in [49]. Fluorescence intensity analyses were performed using ImageJ software (NIH, Bethesda, MD, USA) as following described. Briefly, ten field images from a single coverslip were randomly selected for three coverslips and registered for each experimental condition identifying distinct cells by Hoechst 33342 nuclear staining. Then, individual cell total area was selected using area selection tool and fluorescence intensity value was measured subtracting background. The obtained mean value was used to compare experimental groups.

### 4.10. Tube Formation Assay

A 24-well plate was coated with Matrigel (Becton Dickinson Labware, Franklin Lakes, NJ, USA) mixed to EGM-2 1:1 on ice and incubated at 37 °C for 30 min to allow gelation to occur. As reported in [47], HUVEC cells were added to the top of the gel at a density of 2 × 10^4^ cells/well in the presence or not of the indicated treatments. Cells were incubated at 37 °C with 5% CO_2_. After 12 hours, pictures were captured using EVOS^®^ light microscope (10×) (Life technologies Corporation, Carlsbad, CA, USA). The length of each tube was measured and the number of branches was calculated using ImageJ (NIH, Bethesda, MD, USA) (Angiogenesis Analyzer for ImageJ) software.

### 4.11. Statistical Analysis

Data analyses and statistical evaluations were carried out using Microsoft Excel; the number of independent experiments and *p*-values are indicated in the figure legends. All results are the mean ± standard deviation of at least 3 experiments performed in triplicate. Statistical comparisons between the experimental points were made using two-tailed *t*-test comparing two variables. Differences were considered significant if *p* < 0.05, *p* < 0.01 and *p* < 0.001.

## 5. Conclusions

In the present study we confirmed the ability of ANXA1 to participate in PC progression. The novelty we highlighted is based on the contribution of ANXA1 in PC EVs biogenesis as a further mechanism by which the protein can influence the tumour cell behaviour. Our data suggest the formation of a loop between ANXA1 and the PC EVs which is involved in metastatization. Furthermore, this loop seems to be important also in the autocrine and paracrine modulation of PC cell motility and in regulating the cross-talk between these cells and surrounding tissues, mainly thee endothelial one. Since EVs represent a pivotal element to evaluate PC degree, our work makes reasonable to consider ANXA1 as a co-target to improve prognostic/therapeutic approach.

## Supplementary Materials

Supplementary materials can be found at https://www.mdpi.com/1422-0067/19/12/3878/s1. **Figure S1:** Effects of SS, EDS and EVs fractions from WT MIA PaCa-2 and ANXA1 KO MIA PaCa-2 on themselves in Wound-Healing (**A**) and invasion (**B**) assays as technical controls. * *p* < 0.05, ** *p* < 0.01, *** *p* < 0.001 vs. untreated control; ### *p* < 0.001 for ANXA1 KO MIA PaCa-2 cells vs. WT ones, §§§ *p* <0.001 for ANXA1 KO cell supernatants vs. WT MIA PaCa-2 ones, $$$ *p* < 0.001 for ANXA1 KO MIA PaCa-2 EDS vs. WT MIA PaCa-2 EDS and °° *p* < 0.01 for EVs secreted by ANXA1 KO MIA PaCa-2 vs EVs from WT MIA PaCa-2. **Figure S2:** Immunofluorescence analysis on WT MIA PaCa-2 cells to detect: ANXA1 (panels a-e), F-actin (panels f-j), vimentin (panels k-o), CK8 (panels p-t), CK18 (panels u-y), lamin A/C (panels z-d’) and tubulin (panels e’-i’). Cells were treated or not with SS and EDS fractions from WT and ANXA1 KO MIA PaCa-2. Bar = 10 µm. **Figure S3:** Immunofluorescence analysis on ANXA1 KO MIA PaCa-2 cells to detect: ANXA1 (panels a-e), F-actin (panels f-j), vimentin (panels k-o), CK8 (panels p-t), CK18 (panels u-y), lamin A/C (panels z-d’) and tubulin (panels e’-i’). Cells were treated or not with SS and EDS fractions from WT and ANXA1 KO MIA PaCa-2. Bar = 10 µm. **Figure S4:** Fluorescence intensity for ANXA1, F-actin, vimentin, CK8, CK18, lamin A/C and tubulin signals using ImageJ software. The measurements are determined on ten field images from a single coverslip and randomly selected for three coverslips. **Figure S5:** (**A**) Viability assays on HUVEC cells treated or not with sup (complete cell supernatant, containing FBS), SS and EDS from WT and ANXA1 KO MIA PaCa-2 cells. Results of Wound-Healing assay (**B**) and analysis of invasion speed (**C**) of HUVEC cells treated or not with sup, SS and EDS from WT and ANXA1 KO MIA PaCa-2 cells. (**D**) Representative images of tube formation by HUVEC cells seeded for 12 h on matrigel: EBM-2 1:1 and treated or not with sup, SS and EDS from WT and ANXA1 KO MIA PaCa-2 cells. Analysis of tube length and number of branches calculated by ImageJ (Angiogenesis Analyzer tool) software. Data represent the mean of three independent experiments ± standard deviation with similar results, *** *p* < 0.001 vs. untreated control and °°° *p* < 0.001 for EVs secreted by ANXA1 KO MIA PaCa-2 vs EVs from WT MIA PaCa-2.
