# Correction: Pessolano et al. Annexin A1 May Induce Pancreatic Cancer Progression as a Key Player of Extracellular Vesicles Effects as Evidenced in the *In Vitro* MIA PaCa-2 Model System. *Int. J. Mol. Sci.* 2018, *19*, 3878

**DOI:** 10.3390/ijms25179349

**Published:** 2024-08-29

**Authors:** Emanuela Pessolano, Raffaella Belvedere, Valentina Bizzarro, Paola Franco, Iolanda De Marco, Amalia Porta, Alessandra Tosco, Luca Parente, Mauro Perretti, Antonello Petrella

**Affiliations:** 1 Department of Pharmacy, University of Salerno, via Giovanni Paolo II 132, 84084 Fisciano, Italy; epessolano@unisa.it (E.P.); rbelvedere@unisa.it (R.B.); vbizzarro@unisa.it (V.B.); aporta@unisa.it (A.P.); tosco@unisa.it (A.T.); lparente@unisa.it (L.P.); 2 Department of Industrial Engineering, University of Salerno, via Giovanni Paolo II 132, 84084 Fisciano, Italy; pfranco@unisa.it (P.F.); idemarco@unisa.it (I.D.M.); 3 The William Harvey Research Institute, Barts and The London School of Medicine and Dentistry, Queen Mary University of London, London EC1M 6BQ, UK; m.perretti@qmul.ac.uk

## Error in Figure

In the original publication [1], there was a mistake in Figures 2B and S3 (panel c’) as published. In Figure 2B, three of the four panels were mistakenly taken from the same well. In Figure S3, an image produced for internal controls was erroneously saved and used. The corrected Figure 2B and Figure S3 appear below. The authors state that the scientific conclusions are unaffected. This correction was approved by the Academic Editor. The original publication has also been updated.

## Figures and Tables

**Figure 2:**
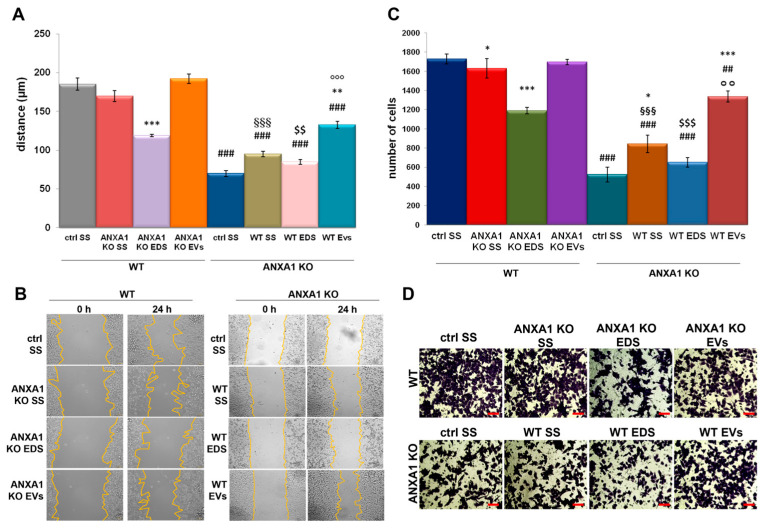
EVs released by WT and ANXA1 KO MIA PaCa-2 affected cell migration and invasion rate. Effects of starved supernatants (SS), EDS, and EVs fractions from MIA PaCa-2 and ANXA1 KO MIA PaCa-2 on WT and ANXA1 KO recipient cells and viceversa in Wound-Healing (**A**) and invasion (**C**) assays. Representative images for migration and invasion assays are reported in (**B**) and (**D**), respectively. Bar = 50 µm. Statistical significance was calculated using *t*-test, * (asterisk) *p* < 0.05, ** *p* < 0.01, *** *p* < 0.001 treated cells vs untreated controls; ## *p* < 0.01 and ### *p* < 0.001 for each point of ANXA1 KO MIA PaCa-2 cells vs WT one, §§§ *p* < 0.001 for the experimental points treated with ANXA1 KO cell SS vs WT MIA PaCa-2 one, $$ *p* < 0.01, $$$ *p* < 0.001 for cells treated with ANXA1 KO MIA PaCa-2 EDS vs WT MIA PaCa-2 EDS and °° (circlets) *p* < 0.01 and °°° *p* < 0.001 for cells in presence of EVs secreted by ANXA1 KO MIA PaCa-2 vs EVs from WT MIA PaCa-2.

**Figure S3:**
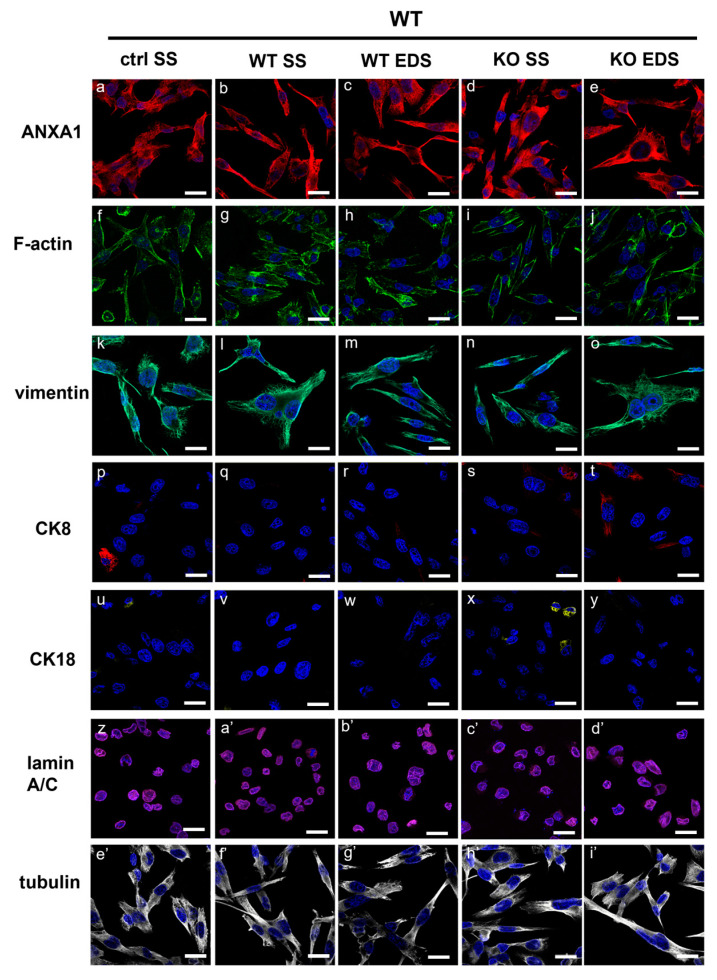
Immunofluorescence analysis on ANXA1 KO MIA PaCa-2 cells to detect ANXA1 (panels **a**–**e**), F-actin (panels **f**–**j**), vimentin (panels **k**–**o**), CK8 (panels **p**–**t**), CK18 (panels **u**–**y**), lamin A/C (panels **z**–**d’**), and tubulin (panels **e’**–**i’**). Cells were treated or not with SS and EDS fractions from WT and ANXA1 KO MIA PaCa-2. Bar = 10 µm.

## References

[1] Pessolano E., Belvedere R., Bizzarro V., Franco P., De Marco I., Porta A., Tosco A., Parente L., Perretti M., Petrella A. (2018). Annexin A1 May Induce Pancreatic Cancer Progression as a Key Player of Extracellular Vesicles Effects as Evidenced in the *In Vitro* MIA PaCa-2 Model System. Int. J. Mol. Sci..

